# Development and Utilization of Corn Processing by-Products: A Review

**DOI:** 10.3390/foods11223709

**Published:** 2022-11-18

**Authors:** Yan Jiao, Hao-Dong Chen, He Han, Ying Chang

**Affiliations:** College of Food and Bioengineering, Qiqihar University, Qiqihar 161006, China

**Keywords:** corn processing, by-products, development, utilization

## Abstract

As an important food crop, corn has an important impact on people’s lives. The processing of corn produces many by-products, such as corn gluten meal, corn husk, and corn steep liquor, which are rich in protein, oil, carbohydrates, and other nutrients, all of which are inexpensive. Their accumulation in large quantities during the production process not only results in a burden on the environment but also the loss of potentially valuable food materials that can be processed. In fact, the by-products of corn processing have been partially used in functional foods, nutrients, feed, and other industries. There is no doubt that the secondary utilization of these by-products can not only solve the problem of waste pollution caused by them, but also produce high value-added products and improve the economic benefits of corn. This paper describes in detail the processing and higher-value utilization of the five main by-products: corn gluten meal, corn husks, corn steep liquor, corn germ, and fuel ethanol by-product. The utilization status of corn processing by-products was discussed roundly, and the development trend of corn processing by-products in China and other countries was analyzed, which provided the reference for the development of the corn deep processing industry.

## 1. Introduction

In a world with an ever-growing population, it has become crucial to use raw materials in a green, environmentally friendly, and repeatable way. The increasing demand for quantity and quality of food will inevitably lead to the production of more waste and by-products during processing. Nowadays, health and development are the common ideals and goals of human beings. People’s lives are gradually changing to healthy and sustainable development. Reducing food waste and improving food utilization has become a mainstream phenomenon in society [1]. The European Union (EU) has planned a circular bioeconomy, an action plan that includes an approach based on reducing, reusing, recovering, and recycling materials and energy [2]. The agricultural and food processing sectors generate a large amount of waste each year [3], so it is necessary to process these agricultural wastes effectively to reduce the negative impact on the environment. Moreover, these agricultural wastes can be used as a cheap source of protein, carbohydrates, and dietary fiber. In particular, the processing by-products of grains or beans are also rich in many nutrients, they can effectively provide some nutritional products for human consumption, and have promising applications in the food industry. However, agricultural by-products are generally considered low-value assets in the post-harvest phase, with about 14% of food loss and waste occurring worldwide [4].

**Figure 1:**
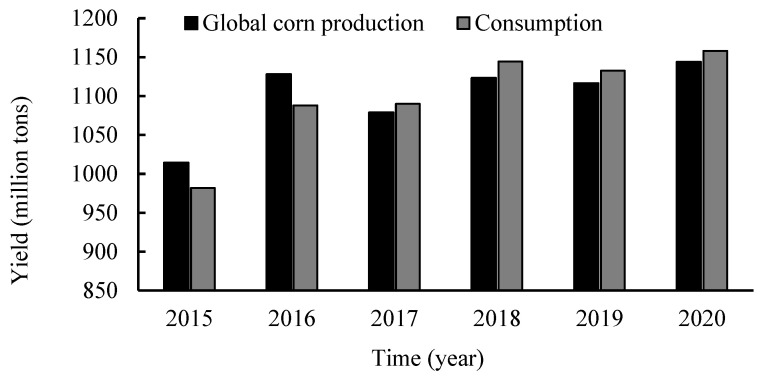
Map of global corn production.

Corn (*Zea mays* L.), which is native to the Americas and now widely distributed in the United States, China, Brazil, and other countries, is an important food crop in the world [5]. It is mainly composed of starch, protein, and fat, which are rich in trace nutrients such as vitamin A, vitamin E, and the trace element selenium. From 2016 to 2020, global corn production and consumption were both above 1000 million tons (Figure 1). Corn is made up of the seed coat, cotyledon, and germ, with different chemical compositions. During processing, corn is broken down into main starch products and by-products. In China, corn by-products are mostly used as low economic value animal feed, which is very wasteful. For example, corn gluten meal contains 60% protein and is a valuable source of protein [6]. Corn husks are rich in oils and dietary fibre [7], corn pulp is rich in mineral elements [8], and each corn by-product has the potential to be used as a functional food component with antioxidant, anti-aging, and anti-obesity properties and can be obtained through secondary processing.

At present, there are more than 1000 kinds of corn by-products processing products, which are widely used in food and chemical industries, fermentation, and other fields. Since the reform of China’s national corn purchasing and storage system in 2016, China’s corn by-product processing industry has developed rapidly. In 2018, China’s production of starch, lysine, monosodium glutamate, and maltitol accounted for 52%, 60%, 68%, and 85% of the world’s total production, respectively, with the export volume of monosodium glutamate, lysine, and citric acid ranking first in the world [9].

According to the different processing degrees of corn raw materials, corn processing can be divided into primary processing and deep processing of by-products. The initial processing of corn involved simple processing such as cleaning, soaking, crushing, separating, and dehydrating. The deep processing of corn by-products is the process of using the first processed corn product as the raw material, and then using external technology to perform secondary processing on it and transform it into the final product. From a single primary corn product to the production of more and more corn derivatives and its by-products, the processing varieties of corn have been continuously enriched, the diversification of the processing of corn by-products has been realized, and the industrial chain has been further extended.

In order to provide a reference for promoting the sustainable development of corn processing industry, this paper summarizes the research and utilization of corn processing by-product in recent years, and the new industrial uses and nutritional products of these corn by-products are introduced, which not only help to improve the economic benefits of corn, reduce the economic burden of farmers [10], but also reduce pollution and contribute to environmental protection. In addition, this paper also aims to discuss the recent advances in further processing and reuse of corn-industry by-products [11]. 

## 2. Corn Gluten Meal

According to research reports, about 180 kg of corn gluten meal (CGM) can be produced for every ton of corn starch syrup processed. The protein content of CGM is as high as about 60%, mainly consisting of zein, globullins, and glutellins [12,13], all of which are very high-quality plant protein sources. CGM contains a high percentage of hydrophobic amino acids, and the rest of the composition is mainly water, fiber, and fat [14,15]. At present, the deep processing products of CGM are described in the following paragraph.

### 2.1. Corn Peptide

CGM is rich in Glu, Leu, Pro, Ala, Phe, and Asp, and its nutritional value is very rich. However, it has low water solubility and is deficient in essential amino acids such as Lys, His, and Trp, considerably, which limits its nutritional value and its direct application as a food ingredient [16]. In order to further expand the application of CGM in the field of food and health care, it can be modified by enzymatic hydrolysis to prepare small molecule oligopeptides, which have better physiological activity and functional properties [17]. In recent years, research has shown that small-molecule oligopeptides have a beneficial effect on human digestion, absorption, and metabolism [18]. Corn peptides (CPs) are usually small molecular peptide fragments produced by the enzymatic hydrolysis of corn gluten powder under the action of proteases [19]. CPs are usually composed of 2–20 amino acids, and the relative molecular weight is between 300–1000 Da. CPs have the characteristics of easy digestion and absorption, and have shown good application prospects in the fields of food and medicine [20]. In 2010, China’s Ministry of Health approved it as a new resource for food [21]. 

There are many methods for preparing CPs, including enzymatic hydrolysis, microbial fermentation, and chemical synthesis [22]. At present, the more commonly used is enzymatic hydrolysis, which includes single and compound enzymatic hydrolysis. The optimization of enzymatic hydrolysis process for corn gluten meal has been relatively mature. The single enzymatic method mainly refers to the use of alkaline protease, neutral protease, or pepsin by optimal conditions for different catalytic reactions of different enzymes. Compound enzyme refers to the interaction and matching ratio between different enzymes, which optimizes the best ratio method, which is more convenient and efficient than single enzyme digestion, and the yield is also higher.

CPs have antioxidant activity, are anti-hypertension, and prevent alcohol injury, and have other unparalleled physiological functions [23]. Ma et al. found that CPs can effectively reduce the amount of ethanol in the blood after alcoholic intake by plasma alanine and leucine [24]. CPs have been found to have the potential ability to promote alcohol metabolism by activating the liver alcohol dehydrogenase (ADH), which leads to a decrease in blood alcohol concentration [25]. In addition, Wu et al. have reported the protective effect of CPs at a dose of 4 g/day may protect the alcoholic liver injury by regulating lipid metabolism and human oxidative stress responses [26].

In the field of food processing, CPs can improve antioxidant capacity of the foods without any decay in other quality parameters. CPs are rich in hydrophobic amino acids, which can promote the secretion of glucagon and can also be used to make sports drinks and other high-protein beverage products [27]. In addition, adding CPs to fresh milk fermented by probiotics and lactic acid bacteria can significantly improve the viscosity, flavor, taste, and health functions of the fresh milk. CPs have extensive application in the field of health food, and because CPs contain a high proportion of alanine and leucine, the concentration of alanine and leucine in the blood increases, which can enhance the alcohol dehydrogenase and acetaldehyde in the liver. The activity of dehydrogenase promotes the decomposition and metabolism of ethanol in the body to achieve the function of sobering alcohol. For this reason, CPs have been widely developed as foods and beverages with functions such as being anti-alcohol and offering liver protection [28,29]. Secondly, the content of leucine and alanine in CPs are high level and play a key role in the anti-fatigue effect, therefore, CPs have been added in sports food to improve muscle resistance to fatigue, there are functional foods that combine CPs with other biologically active substances in the market, and functional foods made of compound substances have the functions of being anti-fatigue, lowering blood pressure, and promoting anti-aging [30]. The main component, technology method, function, and major findings of corn gluten meal are summarized in Table 1.

### 2.2. Zeaxanthin

Zeaxanthin is an important carotenoid derivative, which was first found in corn. Zeaxanthin (3,3′-dihydroxy-β-carotene) is a polyene molecule containing nine alternating conjugated double and single carbon bonds, with both ends of the carbon skeleton connected to a hydroxyl ionone ring. The 3′ chiral carbon atoms in both rings allow for three possible stereoisomers of zeaxanthin, including (3S, 3′S)-zeaxanthin, meso-zeaxanthin and (3R, 3′R)-zeaxanthin. The molecular formula of zeaxanthin is C_40_H_56_O_2_ and the molecular weight is 568.88 Da [31]. Zeaxanthin and lutein are a pair of isomers, and the only difference is that the position of the double bond in the ionone ring is different. Zeaxanthin and lutein are widely found in human eyeballs [32], the pancreas, liver, and other tissues and organs that play an important role in human health [33,34]. Zeaxanthin and lutein are concentrated in the macular area of the central retina of the human eye. The human body cannot synthesize zeaxanthin and lutein, which must be consumed through diet [35]. In CGM, the content of zeaxanthin is about 0.20–0.37 mg/g [36].

Zeaxanthin in the CGM currently follows two extraction methods: (1) The organic solvent method, which can be used to extract zeaxanthin from CGM in organic solvents by utilizing the property of zeaxanthin dissolved in organic solvents. In consideration of the safety of organic solvents, ethanol is mostly selected as the extraction solvent of zeaxanthin. (2) Ultrasonic microwave extraction method. Through ultrasonic oscillation, air-conditioning effect, mechanical effect, thermal effect, etc., the internal structure and state of CGM are changed to increase the penetration of organic solvents into the cell wall, thereby enhancing the extraction efficiency [37].

Zeaxanthin has strong antioxidant and blue light absorbing properties, and is often added as a natural food additive to margarine, butter, beverages, meat, and egg products for the prevention and treatment of eye diseases such as age-related macular degeneration (AMD) and cataracts [38,39,40]. Zeaxanthin is the main macular pigment in the retina of the human body. It absorbs high-energy blue light energy through its own antioxidant activity, thereby preventing the occurrence of human retinal damage [41,42,43]. Zeaxanthin also has a certain anti-cataract effect, and cataracts is the main cause of vision loss in the elderly, as the lens in the eye become turbid. Studies have found that zeaxanthin can prevent cataracts by inhibiting oxidative damage [44]. In addition, because two six-membered carbon rings of zeaxanthin contain one oxygen-containing group, the chemical structure of zeaxanthin has greater stability, with strong coloring ability, and it is widely used in food additives and feed additives [45,46]. In addition, the molecular structure of zeaxanthin shows that there are 11 conjugated double bonds, which enable it to block the chain transmission of free radicals, thus having strong antioxidant activity, and it is often developed as a functional product.

## 3. Corn Husks

Corn husks are the most abundant and least valuable by-product of corn industrial processing, accounting for 10% to 14% of the total fiber content of corn [47]. Corn husks contain 382 g cellulose, 445 g hemicellulose, 66 g lignin, 19 g protein, and 28 g ash per kg of dry matter [48]. Because it is rich in arabinoxylan (70%), it is used to produce xylo-oligosaccharides and dietary fiber. In addition, corn husks are rich in phenolic acids, 90% of which are ferulic acid, which is mainly distributed in the cell walls of aleurone and pericarp layers. Ferulic acid in corn husks exists in free, soluble, and insoluble forms. With a ratio of 1:10:1000, ferulic acid has a strong antioxidant capacity, which can regulate the oxidation state of cells and prevent biological macromolecules such as DNA and proteins from oxidative damage. Compared with the husks of grains such as rice, wheat, and sorghum, the content of phytic acid in corn husks that affects human mineral metabolism is relatively small [49]. Untreated corn husk has no significant beneficial effect on the human health, and excessive consumption of corn husk often leads to human gastrointestinal discomfort because of its insoluble dietary fiber. Therefore, the deep processing of husks can increase the nutritive and economic value of corn. The main processed products of corn husk by-products are as follows.

### 3.1. Carbohydrate

The main component, technology method, function, and major findings of corn husks are discussed in Section 3.1, Section 3.2 and Section 3.3 and summarized in Table 2.

#### 3.1.1. Dietary Fiber

In the middle of the 20th century, the term “dietary fiber” was first used by Hipsley to refer to plant components that resist being decomposed by endoenzymes secreted by mammalian cells [50].

There are about 60% to 70% corn dietary fiber in corn husks, which can be widely used in functional food and medical fields. Dietary fiber has irreplaceable physiological functions in the human body, such as increasing satiety, slowing the rise of blood sugar, and maintaining the integrity of the intestinal mucosa. In addition, dietary fiber can improve human intestinal flora and provide energy and nutrition for the growth and reproduction of human probiotics [51]. Studies have shown that dietary fiber can help reduce the concentration of postprandial blood glucose, insulin, and triglyceride, reduce the concentration of cholesterol in human blood, and plays an important role in people’s health. The recommended daily dietary fiber intake is 28 g/day for adult women and 36 g/day for adult men [52].

As a kind of plant tissue, dietary fiber has a strong water absorption capacity, and corn dietary fiber has a water absorption capacity of 1.5 g water/g powder, so it can accelerate the transportation speed of human digestive tract feces, can increase the volume of feces, effectively clean the intestines, and reduce the pressure of the rectum and urinary system [53]. In addition, the number of dietary fibers can improve intestinal flora and increase the number of probiotic bacteria such as *Lactobacillus* and *Bifidobacterium* to prevent a series of gastrointestinal diseases, such as constipation and hemorrhoids, etc. [54]. At present, the treatment of diabetes and hypertension still lacks specific drugs, and treatment requires a reasonable diet. Dietary fiber can effectively reduce blood sugar, blood pressure, and blood lipids [55,56,57]. Dietary fiber has strong binding and exchange ability with cations, which can exchange with sodium ions and potassium ions in the intestinal tract, and promote the excretion of Na+ and K+ ions with urine and feces, thus reducing the ratio of Na+ and K+ ions in the blood, which directly play the role of lowering blood pressure [58]. Dietary fiber absorbs glucose, reduces the absorption of glucose by the human body, effectively reduces the content of glucose in the blood, and has the effect of treating diabetes [59]. 

Dietary fiber has good physical and chemical properties such as water retention, oil retention, emulsification and gel properties, and more functional properties for human health. In recent years, studies have shown that adding soluble dietary fiber to bread can effectively inhibit short-term degradation of bread and improve the stability of bread [60]. Adding dietary fiber into beverages can improve the stability and dispersion of the beverage. Adding dietary fiber to meat products can maintain the moisture of meat products, increase the viscosity and gel capacity of meat products, and inhibit the growth of microorganisms [61]. Corn dietary fiber, which consists of cellulose and hemicellulose, is one of the most important sources of dietary fiber in grains and was once discarded or used as animal feed. In addition, corn dietary fiber can also absorb nitrites, significantly reducing the risk of cancer [62]. Treatment of corn bran dietary fiber with xylanase increased its ability to bind bile salts in vitro [63]. Corn dietary fiber contains many potentially valuable components such as cellulosic fiber gel, corn fiber gel, corn fiber gum, xylo-oligosaccharides, corn fiber oil, and ferulic acid [64].

**Table 2:** The main component, technology method, function, and major findings of corn husks.

Component	Technology Method	Function	Major Findings	Reference
Dietary fiber	At present, dietary fiber extraction is mainly based on chemical preparation methods, including membrane separation, enzymatic hydrolysis, and fermentation	Dietary fiber has strong binding and exchange ability with cations, and absorbs glucose	Can exchange with sodium ions and potassium ions in the intestinal tract, and promote the excretion of Na+ and K+ ions with urine and feces	[58]
Polysaccharides	Polysaccharides in corn husks are usually extracted by acid, base or organic solvent, enzyme assisted extraction, ultrasonic assisted extraction, and other methods	Polysaccharides have significant functional activities such as immune regulation, anti-oxidation, anti-tumor, and blood pressure lowering functions	Polysaccharides can improve immunity by affecting the morphology, phagocytosis, cytokine secretion, and other aspects of macrophages or by increasing the volume of macrophages	[65]
Protein	By the ethanol extraction and alkali acid sinking method	Main Protein in corn husk is zein. Zein contains hydrophobic and hydrophilic groups, which had good amphiphilicity	The zein protein is quickly extracted from the ethanol solution, and the zein protein particles will bind together to form a fibrous structure, and degradable zein film can be formed	[66]
Corn husk oil	The traditional preparation methods of corn husk oil mainly include the pressing method and organic solvent extraction method.	Corn husk oil is also rich in phytosterols, which play a critical role in maintaining cholesterol balance, anti-oxidation, and anti-aging in the human body.	They are the main components of cell membranes and precursors of vitamin D and various hormones. Since the human body cannot synthesize sterols by itself, food is the only source of sterols.	[67]

#### 3.1.2. Polysaccharides

Polysaccharides are polymeric carbohydrate macromolecules composed of long chains of monosaccharide units joined by glycosidic linkages. There are two types of polysaccharides: homo-polysaccharides and hetero-polysaccharides; homo-polysaccharides have only one monosaccharide repeated in the chain, and hetero-polysaccharides are composed of two or more monosaccharides [65]. With the development of molecular biology, the scientific community has gradually realized that polysaccharides with proteins are extremely important biomacromolecules and play an important role in the growth and development of organisms. 

Polysaccharides in corn husks are usually extracted by water extraction combined with ethanol precipitation. Because polysaccharide molecules contain a large number of polar groups with good water solubility, the higher the temperature, the better the solubility of the polysaccharides. This method has mild reaction conditions and a simple operation, but the extraction cycle is too long and the extraction efficiency is low. Therefore, acid, alkali, or organic solvent extraction is usually used, as well as enzyme-assisted extraction, ultrasonic-assisted extraction, microwave extraction, etc. [66,67]. Polysaccharides are one of the main functional components of corn husks, which have significant functional activities such as immune regulation, anti-oxidation, anti-tumor, and blood pressure lowering functions [68]. For example, polysaccharides can improve immunity by affecting the morphology, phagocytosis, cytokine secretion, and other aspects of macrophages or by increasing the volume of macrophages. It can also enhance the secretion of immunoglobulin and cytokines by promoting the proliferation of lymphocytes [69]. Polysaccharides can reduce the chain length of lipid peroxidation and prevent or slow down the process of lipid peroxidation. Direct removal of reactive oxygen species can be achieved by capturing various reactive oxygen species in a chain reaction of lipid peroxidation. In addition, polysaccharides can also act as free radical scavengers by pairing with their single electrons so that their absorption gradually disappears, thus exerting their antioxidant power [70,71,72]. Corn polysaccharides also have a significant effect on lowering blood sugar, and corn colysaccharides are composed of xylose and arabinose, which is connected by β-1,4 glycosidic bonds. Xylose and arabinose can be obtained by hydrolysis of dilute sulfuric acid. Among them, L-arabinose is a five-carbon aldose, which can be used as a low-calorie sweetener without decomposition. Generating heat can inhibit the enzymes that hydrolyze disaccharides, block the metabolism of sucrose, and then have a significant inhibitory effect on the increase in blood sugar [73]. Black glutinous corn polysaccharides (BGCP) were isolated and the hypoglycemic activity of alloxan-induced diabetic mice was evaluated. In the study byZhang et al., mice were given 800, 500 or 200 mg/kg BGCP daily for 4 weeks. The results showed that blood glucose levels were reduced in all three groups with the addition of BGCP, with the 800 mg/kg treatment leading to greater hypoglycemic effects than low-dose treatments [74].

### 3.2. Protein

As corn gluten meal, the protein in corn husks is mainly zein. Zein is a safe, non-toxic, renewable, natural, low-cost, biocompatible, and degradable protein, and it is composed of four components (α, β, γ, and δ) with different peptide chains, molecular sizes, and solubility. The most abundant protein in commercial zein is alpha-zein. Zein is soluble in 60–95% ethanol, acetone, and alkaline aqueous solutions with pH ≥ 11.5. In practical application, zein contains hydrophobic and hydrophilic groups, which had good amphiphilicity [75,76,77].

Zein has a unique amino acid composition, which contains a high proportion of hydrophobic amino acids and more sulfur-containing amino acids, but lacks charged acidic, basic, and polar amino acids, which makes it uniquely soluble [78,79].

At present, in view of the difficulty of recycling plastic packaging and the difficulty of degradation, environmental pollution is becoming more and more serious. With the improvement of environmental awareness, people’s demand for biodegradable packaging materials is increasing. The film-forming performance of biological polymer materials has attracted people’s attention. The unique amino acid composition of zein makes it have good film-forming properties. The film-forming forces of zein are mainly hydrophobic bonds, hydrogen bonds, and limited disulfide bonds between proteins. The zein protein is quickly extracted from the ethanol solution, and the zein protein particles will bind together to form a fibrous structure. After the subsequent steps of coating, evaporation, dehydration, and other steps, a moisture-resistant, oxygen-resistant, oil-resistant, and degradable zein film can be formed [80,81].

Zein has been used widely in the field of the food industry, and is mainly used in food embedded agents, food emulsifiers, free gluten-free foods, food packaging materials, and reasonable development. For example, zein has excellent viscoelasticity as a substitute for gluten protein [82]. Zein can also encapsulate fat-soluble functional active substances, pigments, or flavor components. By encapsulating and protecting the centrally active substances, it can effectively improve the stability and partial functional properties of the active ingredients, and ensure the nutritional value of the centrally active substances. At the same time, the adhesion of zein can prolong the residence time of the drug on the surface of the intestinal mucosa, realize the enteric release of the central substance, and promote the absorption and utilization of the central substance. When fatty acids are added to zein solutions, they can be used as binders for food materials or wood, metal, and other materials. Zein contains a high content of branched chain amino acids and neutral amino acids, which can be used to prepare functional peptides with low molecular weight and high activity, such as high F-value oligopeptides and antioxidant peptides, which can be used in biological medicine and functional health products. Reasonable development and utilization of zein will bring huge economic benefits to the food industry [83].

### 3.3. Corn Husk Oil

There is about 5–12% oil in corn husks, and these oils contain more than 80% unsaturated fatty acids, which play an important role in the development of human brain function and the metabolism of human blood lipids, which can improve human immunity and prevent the risk of prostate and other diseases [84,85]. In addition, phytosterol is a kind of natural plant active substance with a similar structure to cyclic alcohol, which can be divided into the free type and the esterified type. Phytosterols widely exist in various vegetable oils, seeds, and nuts. They are distributed in the roots, stems, leaves, fruits, and seeds of plants. They are the main components of cell membranes and precursors of vitamin D and various hormones. Since the human body cannot synthesize sterols by itself, food is the only source of sterols. Corn husk oil is also rich in phytosterols, which play a critical role in maintaining cholesterol balance, anti-oxidation, and anti-aging in the human body. Numerous epidemiological studies have proven that phytosterols contribute to the incidence of chronic diseases inversely.

Corn husk oil is difficult to extract because of the tight structure of the husk. Some scholars have used ultrafine pulverization and dilute acid hydrolysis to improve the yield of corn husk oil, but the effect is not effective. The traditional preparation methods of corn husk oil mainly include the pressing method and organic solvent extraction method. For the subcritical extraction solvent, according to the physical properties of similar phase dissolution of organic matter, counter current extraction of materials is carried out through the molecular diffusion process between materials and the extraction solvent in the contact process, and then the extraction solvent is separated from the extracted target component through the process of decompression evaporation. This method is non-toxic, harmless, and pollution-free, and does not destroy the advantages of biological activity of the product, and can be effectively used to extract corn oil from corn husk, but can also reduce the production cost in the whole process of corn development, improving the comprehensive economic benefits of the corn processing industry [86].

## 4. Corn Steep Liquor

The first step of corn deep processing is to foam the corn, which will produce a large amount of soaking water. In order to break the -S-S- bond in the protein, the protein network in the corn kernel is broken in order to release the wrapped starch. The corn kernels should be soaked in an aqueous solution of sodium bisulfite first, and the viscous liquid obtained by concentrating the soaking solution is corn steep liquor (CSL) [87]. China is a big producer of corn starch. Most cornstarch on the domestic market is produced by a wet process, and a large amount of CSL will be produced during the production process. CSL contains a lot of protein, soluble sugar, and sulphide compounds; at present, corn steep liquor has not been used effectively, but is directly discarded, which not only increases the production cost of corn starch, and causes a lot of waste of resources, but also has a great impact on the development of the corn industry and causes environmental pollution and destroys the ecological environment. Therefore, the development of comprehensive utilization of CSL is of great significance to the recovery and secondary development of corn steep liquor, which is of great significance for increasing the added value of corn industry products and reducing environmental pollution.

CSL is the main by-product of corn starch production by the wet process. It is a viscous, acidic slurry with an aromatic smell and yellowish-brown color. It is considered a rich and cheap source of carbon, nitrogen, amino acids and minerals, and has a broad application prospect in the development of the biological process. Some studies have shown that using CSL as a nitrogen source to produce microbial metabolites (such as citric acid, amylopectin, enzyme, and bioenergy) has a very good effect [88]. The crude protein content of CSL is approximately 20% and the solids content is approximately 50%. Therefore, it can be used as an excellent animal feed ingredient; in addition, CSL is rich in sucrose, nutrients, and elements such as Ca, Mg, Al, Fe, Mn, Mo, P, and S, which provide a good source of organic nitrogen and carbon for microbial growth [89]. Selim, MT et al. studied the Lactic acid concentration of about 44.6 g/L with a high yield (0.89 g/g) obtained using 60 g/L of CSL sugar, inoculum size 10% (*v*/*v*), 45 degrees C, and sodium hydroxide or calcium carbonate as a neutralizing agent [90]. Biosurfactants extracted from CSL can be used in dairy production. These results demonstrate that adding this biosurfactant to drinkable yogurt can promote the growth of Lactobacillus casei, which is considered to be a probiotic bacterium [91]. Phytic acid, also known as inositol hexaphosphate, can bind with calcium ions to form calcium phytate, which has a very good therapeutic effect on gastritis, diarrhea, and rickets. The extraction of plant calcium from CSL has a simple operation, a short cycle, and is low cost. At present, the technology for extracting calcium phytate from corn steep liquor is relatively mature [92,93].

## 5. Corn Germ

Corn kernels are composed of the seed coat, endosperm, and embryo. Corn germ is a part of the corn embryo, which is located below the corn kernel and is mainly involved in the growth and development of corn (Different Strategies to Obtain Corn (*Zea mays* L.) Germ Extracts with Enhanced Antioxidant Properties, Design of corn germ extractor based on S7-1200 PLC). Corn germ is the beginning of the growth and development of corn, and its weight only accounts for about 13% of corn, but it is very rich in nutritional value and rich in a variety of nutrients (Corn germ with pericarp in relation to whole corn: nutrient contents, food and protein efficiency, and protein digestibility-corrected amino acid score), concentrating more than 80% of fat and inorganic salt, 60% of sugar and 20% of the protein in the whole corn, and also contains phospholipids, sterols, and other nutritional ingredients [94]. Cornstarch is industrially separated from corn kernels through a process called dry or wet milling, leaving corn germ as the main residue [95]. Corn germ has been studied as a feed for ruminants due to its desirable nutritional properties [96]. The inclusion of whole corn germ in ruminant diets aims to increase energy density and polyunsaturated fatty acids, to obtain higher levels of conjugated linoleic acid (CLA) in the meat, which are beneficial to human health [97]. The addition of 120 g/kg dry matter corn germ into the diet of lambs did not affect the carcass characteristics, physicochemical composition, and sensory properties of the meat. When corn germ dosage was 76.7 g/kg dry matter, the distribution of polyunsaturated fatty acids in lamb meat was the best [98]. Corn germ can be used at low levels in the diet of broilers without compromising their productive rates [99]. Moreover, the main processed products of corn germ by-products are as follows.

### 5.1. Corn Germ Oil

Corn germ oil is a nutritious and healthy edible oil. It has a transparent golden yellow color and a fragrant fragrance. It is called “liquid gold” by Western countries. Corn germ oil contains 80–85% of unsaturated fatty acids, oleic acid, linoleic acid, and α-linolenic acid. The content of linoleic acid is as high as 56%. Linoleic acid is an essential fatty acid that the human body cannot synthesize by itself, and an important component of human cells. Corn germ oil is also rich in natural vitamin E, which can effectively prevent atherosclerosis and reduce the risk of coronary heart disease. In addition, corn germ oil is rich in phytosterols and phospholipids. Phytosterol is called “the key to life”, and the phytosterol content in corn germ oil is more than all food, up to 633 mg/100 g [100,101,102]. In recent years, more and more people are consuming corn oil, and the physical and chemical characteristics of corn germ oil are listed in Table 3.

At present, there are many extraction methods for corn germ oil, and the traditional extraction method of corn germ oil is the pressing method. The pressing method is to squeeze the oil out through mechanical pressure, which is a method commonly used in oil factories. The process of this method is relatively simple, the supporting equipment is scarce, and the quality of the prepared corn germ oil is good, but its disadvantages are lower oil production efficiency and more serious waste. The “pre-leaching” method is the mainstream process of current edible oil production enterprises. The pre-pressing leaching method is a method in which the corn germ is first pressed, and then the oil is extracted with an organic solvent. Pre-pressing only squeezes about 70% of the fat in the embryo, and the pressing temperature and pressure are lower than those of the full pressing method. Therefore, the crude oil is light in color and high in quality. After pre-pressing, the corn germ press cake is extracted [104]. This method combines the advantages of the two technologies, but there are still many shortcomings. For example, protein denaturation also exists in the pre-pressing process, which affects the quality of oil. Supercritical CO_2_ extraction technology is an emerging extraction technology, which has been widely used in the field of corn germ oil extraction. This method is used to extract corn germ oil efficiently by adjusting the influencing factors in the extraction process by using CO_2_ in the supercritical state. The extraction efficiency of corn germ oil by this method is obviously improved and the quality of corn germ oil is better. The corn germ oil extracted by different methods had some differences in oil yield and the quality of corn germ oil products.

### 5.2. Corn Germ Protein

The protein in corn germ is of a high quality and has high nutritional value. Compared with corn, the content of crude protein, lysine, and methionine of corn germ meal is 2–3, 3.2, and 1.4 times that of corn, respectively and the content of vitamins and minerals is also much higher, so it has a great value of development and utilization [105]. Corn germ protein contains all the essential amino acids needed by the human body and has a high biological value (BV, 64–72%), second only to eggs (94%) and milk (85%). The protein efficacy ratio (PER) of corn germ protein was 2.04 to 2.56, comparable to soy protein (2.32, PER) and casein (2.5, PER) [106]. Among common grains, corn germ protein is one of the best plant protein nutrient sources, and its amino acid composition is in good agreement with the human protein standards recommended by the FAO/WHO. In addition to the rich nutritional value of corn germ protein, it has been reported that the antioxidant activity of its hydrolysate has been studied. The results show that the corn germ protein hydrolysate has strong antioxidant capacity, and has an inhibitory effect on the oxidation of linoleic acid. It has an inhibitory effect on the peroxide value of oils and fats, and has a good development prospect [107].

The proteins in corn germ are mainly albumin and globulin, which have good functional properties. Corn germ protein has good solubility, and its nitrogen solubility index (NSI) is very high under alkaline conditions. Corn germ protein also has better water absorption, much higher than soybean protein. The oil holding capacity of corn germ protein was also better, which was similar to that of soybean concentrate [108]. In addition, corn germ protein also has good emulsification, foaming, and gel properties, and is an excellent functional food raw material or food additive [109]. At present, people mainly focus on the extraction of corn germ oil, and corn germ protein is also a very high-quality protein. The industrialized production of corn germ protein can significantly improve the tissue structure and taste of the target product, increase the yield of the product, and prolong the shelf life.

The extraction method of corn germ protein is mainly the alkaline extraction method, which is a very common method for extracting vegetable protein. The protein is extracted mainly by utilizing the properties of corn germ protein, which is an amphoteric compound, with better solubility under alkaline conditions and less solubility near the protein’s isoelectric point. In the solution of soaking corn germ, an alkaline solution is slowly added, and when the pH of the solution reaches about 9.5, the protein is precipitated, and the corn germ protein can be obtained by centrifugation after standing for a period of time. This method is a very classical extraction method with a simple operation, high extraction rate, and a low cost, but the color of corn germ protein obtained by this method is poor, and the extraction process will affect the physical, chemical properties, and nutritional properties of corn germ protein.

The enzyme-alcohol method is also a common extraction method for corn germ protein, and cellulase and amylase were used to hydrolyze the corn germ to release the protein and fat, and then ethanol was used to extract the protein from the corn germ. The corn germ protein extracted by the method has a good taste, mild reaction conditions, and good specificity, and at the same time, it also avoids environmental pollution when the protein is extracted by the alkali-soluble acid precipitation method, but the cost is relatively high. The reverse micelle technology uses a non-polar organic solvent to dissolve the surfactant. When the concentration is excessive, micelles are formed in the organic solvent. The hydrophilic macromolecules dissolved in the water hole will be extracted in the form of micelles, so that the oil and protein can be separated. The extraction conditions of reverse micelle technology are mild, the solvent can be reused, and large-scale operation can be realized, which has broad industrial development and application prospects. The extraction of corn germ protein by reverse micellar technology can retain the maximum activity of corn germ protein components, and the oil absorption, emulsification, and emulsification stability are relatively excellent [110].

Corn germ protein has good functional properties and nutritional value, and it has been widely used in the food industry [111]. In baked foods, corn germ protein can be used as a good food nutritional additive, and adding corn germ protein can make up for the deficiency of lysine and threonine in wheat protein. With the improvement of people’s living standards, more attention is being paid to the balanced nutrition of the diet. As a high-quality vegetable protein, corn germ protein can be used as a substitute for milk to meet the needs of lactose intolerant people, and because corn germ protein has a better emulsifying ability, no emulsifier needs to be added to the formula. Corn germ protein drink is milky white in color, delicate in taste, and has a special corn flavor. Corn germ protein has good water absorption, oil absorption, and gel properties, and adding it into meat products can increase the water retention and oil retention properties of meat products, reduce fat separation, increase yield, and effectively improve the nutritional value of meat products [112,113]. Corn germ protein can effectively replace traditional protein additives such as casein, whey protein concentrate, and soy protein. Therefore, further in-depth exploration of corn germ protein research in the fields of food, health and medicine will be a future research trend. The main component, technology method, function, and major findings of corn germ are summarized in Table 4.

## 6. Fuel Ethanol by-Product

**Figure 2:**
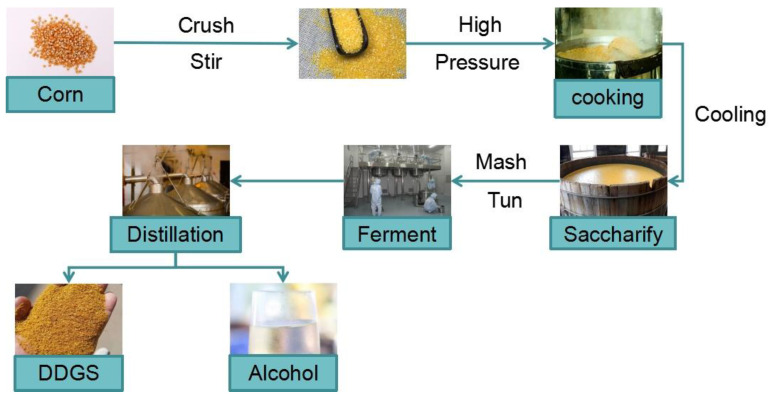
Process flow diagram of ethanol and DDGS production.

Corn distiller grains (DDGS) are produced by a mixed fermentation of corn seeds, selected yeast, and enzymes in a fuel ethanol plant. DDGS is mainly composed of dried distillate (DDG) and soluble concentrate (DDS), which is rich in protein, amino acids, vitamins, and other nutrients. Each 100 kg of corn can produce 34.1 kg of ethanol and 31.6 kg of DDGS. The yield is large but the price is low. Due to the high acidity and high viscosity of corn distillers grains, the COD (Chemical Oxygen Demand) value can reach 30,000–50,000 mg/L, and the BOD (Biochemical Oxygen Demand) value can reach 20,000–30,000 mg/L, and if the waste is directly discharged, it will not only cause serious pollution to the environment but also be a great waste of resources, so it should be fully utilized [114]. Figure 2 is a typical process flow chart of ethanol production by the corn whole grain method.

There is also much research on the application of corn DDGS in livestock and poultry. For example, two groups of Silurus glanis were fed for two consecutive weeks with DDGS in one group and no DDGS in the other. The results showed that the apparent digestibility of corn DDGS is beneficial to Silurus Glanis, and 30% DDGS can be added into the Silurus Glanis’ diet without affecting the growth performance and nutrient utilization of Silurus Glanis [115]. Determination of the effect of different levels of corn DDGS feed on broiler amino acid digestibility showed that higher levels of DDGS could reduce broiler amino acid digestibility [116]. Some scholars have used a combination of alkaline and enzymatic methods to extract cellulose from corn kernels and DDGS. The minimum crude cellulose yield of corn kernels and DDGS is 1.7% and 7.2%, respectively, and the cellulose content is 72% and 81%, respectively. When extracting solids with 35–81% cellulose content, the obtained cellulose can hold up to nine times its weight, so it can be used as an absorbent. Cellulose is also used as paper, composites, lubricants, and nutritional supplements [117].

## 7. The Challenges of Corn Processing by-Products

In recent years, with the continuous development of corn processing projects, corn processing is undergoing technical changes. In order to guarantee the success of the corn processing industry and promote the healthy and stable development of enterprises, it is necessary to strengthen the comprehensive utilization of corn by-products. At present, there are also many technical and process challenges. For instance, modern biotechnology (efficient enzymatic hydrolysis and fermentation technology) and separation and purification technology need to be developed to increase the added value of corn processing products and reduce environment pollution. How do we apply these advanced technologies, reduce costs to develop high-priced products, and reduce the discharge of environmental pollutants such as corn pulp and by-product of fermentation so as to maintain ecological balance is the main challenge we will meet.

## 8. Conclusions

As the demand and nutritional quality of corn products increase, the amount of by-products produced during corn processing also increases. In order to maximize the utilization value of corn, reduce waste of resources, and fully realize the sustainable development of the corn industry, it is necessary to seek reasonable processing and utilization of corn by-products. The high value processing of corn by-products has broad market prospects and huge business opportunities, and it is of great practical significance to strengthen the development of corn by-products processing.

## Figures and Tables

**Table 1:** The main component, technology method, function, and major findings of corn gluten meal.

Component	Technology Method	Function	Major Findings	Reference
Corn peptide	There are many methods for preparing CPs, including enzymatic hydrolysis, microbial fermentation, and chemical synthesis. At present, the more commonly used is enzymatic hydrolysis, and enzymatic hydrolysis is carried out through the optimal conditions for catalytic reactions of different enzymes	CPs have antioxidant activity, anti-hypertension, metabolic alcohol, and other unparalleled physiological functions	CPs can effectively reduce the amount of ethanol in the blood after alcoholic intake by plasma alanine and leucine	[23]
			CPs have been found to have the potential ability to promote alcohol metabolism by activating the liver alcohol dehydrogenase (ADH)	[24]
			CPs may protect the alcoholic liver injury by regulating lipid metabolism and human oxidative stress response	[25,26,27]
		CPs has sobering alcohol function	As CPs contain a high proportion of alanine and leucine, the concentration of alanine and leucine in the blood increases, which can enhance the alcohol dehydrogenase and acetaldehyde in the liver. The activity of dehydrogenase promotes the decomposition and metabolism of ethanol	[28,29,30]
Zeaxanthin	Zeaxanthin in the CGM is currently following two extraction methods: (1) Organic solvent method; (2) Ultrasonic microwave extraction method	The human body cannot synthesize zeaxanthin and lutein, which must be consumed through diet	Zeaxanthin can prevent cataracts by inhibiting oxidative damage	[31]

**Table 3:** Physical and chemical properties of corn germ oil [103].

Property	Value
Iodine value (I_2_ g/100 g)	109–133
Saponification value (KOH mg/g)	180–195
Refractive index (20 °C)	1.4726–1.4759
Fat freezing point (20 °C)/°C	−8–−11
Soap content (%)	≤0.3
Specific gravity (20 °C/40 °C)	0.9152–0.9234

**Table 4:** The main component, technology method, function, and major findings of corn germ.

Component	Technology Method	Function	Major Findings	Reference
Corn germ oil	The traditional extraction method of corn germ oil is pressing; The dominant process is the “preleaching” process; Emerging technology supercritical CO_2_ extraction.	Corn germ oil contains 80–85% of unsaturated fatty acids, oleic acid, linoleic acid, and α-linolenic acid	Linoleic acid is an essential fatty acid that the human body cannot synthesize by itself, and is an important component of human cells. Corn germ oil is also rich in natural vitamin E, which can effectively prevent atherosclerosis and reduce the risk of coronary heart disease.	[100,101,102]
Corn germ protein	The main extraction methods of maize germ protein are alkaline extraction and enzyme-alcohol extraction, whereas reverse micelle technology is a new method of using non-polar organic solvent to dissolve surfactants.	The protein in corn germ belongs to high-quality protein and has high nutritional value.	The corn germ protein hydrolysate has strong antioxidant capacity, and has an inhibitory effect on the oxidation of linoleic acid.	[107]
		Corn germ protein can be used as a good food nutritional additive; adding corn germ protein can make up for the deficiency of lysine and threonine in wheat protein.	Corn germ protein can be used as a substitute for milk to meet the needs of lactose intolerant people, and because corn germ protein has better emulsifying ability, no emulsifier needs to be added to the formula.	[112]

## Data Availability

Data is contained within the article.
